# Alleviation of neuropathic pain by regulating T-type calcium channels in rat anterior cingulate cortex

**DOI:** 10.1186/s12990-015-0008-3

**Published:** 2015-03-06

**Authors:** Feng-Yan Shen, Zhi-Yu Chen, Wei Zhong, Li-Qing Ma, Chong Chen, Zhou-Jing Yang, Wei-Lin Xie, Ying-Wei Wang

**Affiliations:** Department of Anesthesiology and Intensive Care Medicine, Xinhua Hospital, College of Medicine, Shanghai Jiaotong University, 1665# Kongjiang Road, Shanghai, 200092 China; Institute of Brain Functional Genomics, East China Normal University, 3663# North Zhongshan Road, Shanghai, China

**Keywords:** Neuropathic pain, Anterior cingulate cortex, T-type calcium channel, Central sensitization

## Abstract

**Background:**

It has been demonstrated that administration of T-type calcium channel (TCC) inhibitors could relieve the neuropathic pain by intraperitoneally or intrathecally. TCCs are not only expressed in dorsal root ganglia or dorsal horn, but also in some of the pain associated brain regions. In the present study, we sought to investigate whether modulating TCCs in the anterior cingulate cortex (ACC) could alleviate the neuropathic pain.

**Results:**

(1) Ca_v_3.2 was up regulated in rat ACC after chronic constriction injury (CCI). (2) T-type calcium current intensity was increased in CCI animal model. (3) TCC inhibitor reduced miniature excitatory postsynaptic currents frequency of ACC neurons in CCI animal model. (4) TCC inhibitor suppressed the firing rate of ACC neurons in CCI animal model. (5) Both mechanical and thermal allodynia were partially relieved by ACC microinjection with TCC inhibitor.

**Conclusions:**

TCCs in the ACC may be contributing to the maintenance of neuropathic pain, and the neuropathic pain can be alleviated by inhibiting the neuronal activity of ACC through modulating the TCCs.

## Background

Central sensitization is an important mechanism of persistent neuropathic pain. Nociceptive neurons in the dorsal horns of the spinal cord are reported to become sensitized by peripheral tissue damage or inflammation [1]. Further study reveals that pain perception is associated with activity in many parts of the brain, including the thalamus, hypothalamus, midbrain, lentiform nucleus, somatosensory cortices, insula, prefrontal cortex, and anterior and parietal cingulum [2].

The anterior cingulate cortex (ACC) is the frontal part of the cingulum that resembles a “collar” surrounding the frontal part of the corpus callosum. The ACC is involved in rational cognitive functions such as reward anticipation, decision-making, empathy, impulse control, and emotion [3,4]. The ACC is also reported as a key cortical region involved in the persistent neuropathic pain [5,6]. Disruption of long-term potentiation in the ACC can relieve neuropathic pain [7]. Moreover, our previous work has demonstrated that neuronal activity in the ACC is upregulated in animal model of neuropathic pain [8]. These studies suggest that inhibiting the neuronal activity of the ACC may alleviate neuropathic pain.

T-type calcium channels (TCCs) are a type of low threshold voltage-gated ion channel, and they are important for the repetitive firing of action potentials in neurons with rhythmic firing patterns [9]. TCC-mediated burst firing activates cortical neurons more efficiently than tonic firing, potentially providing further amplification of the thalamocortical relay of sensory signals [10-12]. Thus, this neuronal activity may be inhibited by modulating TCCs. We therefore sought to investigate whether the neuropathic pain could be alleviated by inhibiting the neuronal activity of the ACC through modulating the TCCs.

## Results

### The expression of TCC RNA was upregulated in rat ACC after chronic constriction injury

**Figure 1:**
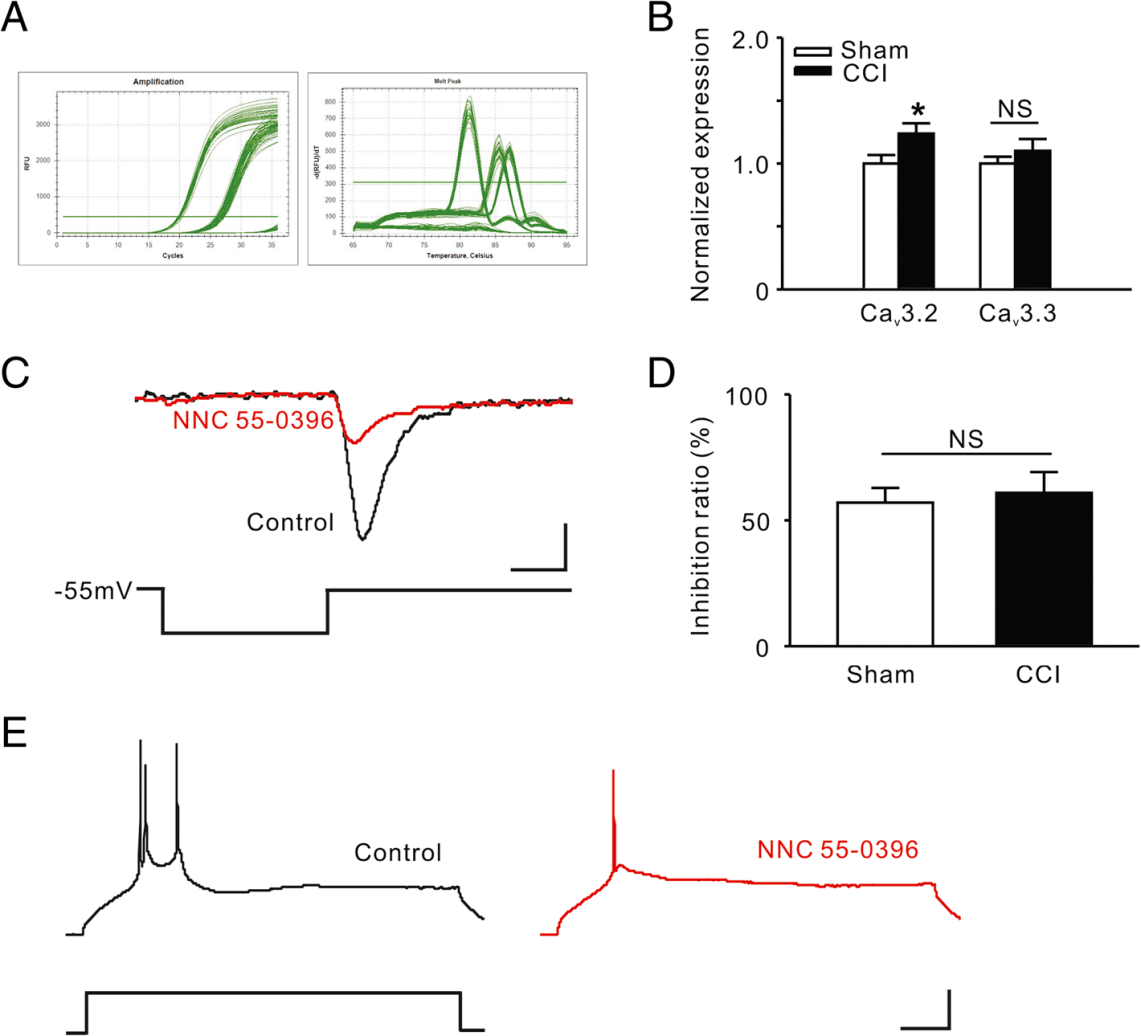
**The expression of TCCs in rat ACC. A**, Quantitative Real-time PCR standard curves and melt culves. **B**, Comparison of RNA levels of TCC subunits between the Sham and CCI groups. *P < 0.05 compared to the Sham group. NS, not significant. **C**, Induction of T-type currents in a pyramidal cell of ACC. Black trace representing the T-type current evoked by transient membrane depolarization, and the red trace representing the attenuated current after *NNC 55–0396* (a selective TCC inhibitor, 20 μM) bath application. Scale bars: 20 pA/35 mV and 25 ms. **D**, Comparison of inhibition ratio of *NNC 55–0396* on T-type currents between the Sham and CCI groups. **E**, TCC mediated burst firing in ACC. Black trace representing a burst firing elicited by a transient current injection, and the red trace representing a single spike elicited by the same current injection after *NNC 55–0396* bath application. Scale bars: 20 pA/25 mV and 100 ms.

Kang et al. have mentioned that TCCs are expressed in mouse ACC [13]. However, no further information about the functions of TCC in the ACC is available from that study or any other reports. TCCs may contain one of three α1 subunits, α1G (Ca_v_3.1), α1H (Ca_v_3.2) or α1I (Ca_v_3.3) [14]. We performed RT-PCR to investigate whether the expression of TCC subunits is changed after chronic constriction injury (CCI). In the current study, the Ca_v_3.1 expression in ACC was too low to be quantifiable. Compared to the sham group, the expression of Ca_v_3.2 was higher in the CCI group (Sham: 1.0 ± 0.07, n = 10 vs. CCI: 1.2 ± 0.08, n = 10, *P* = 0.043 using the normal Student’s *t* test; Figure 1A, B). Meanwhile, there was no significant difference in the expression of Ca_v_3.3 between the two groups. Next we further validated the presence of TCCs by in vitro electrophysiological experiments. We evoked an inward current using the TCC determination methods [15] in ACC pyramidal cells (Figure 1C). Moreover, the current was attenuated by *NNC 55–0396*, a selective TCC inhibitor [16]. However, we found that there was no significant difference in the inhibition ratio between the Sham and CCI groups (Figure 1D). Because the TCC is considered to contribute to the bursting activity, we investigated whether *NNC 55–0396* could affect the bursting. We found that the elicited burst firing was attenuated by the TCC inhibitor (Figure 1E).

**Figure 2:**
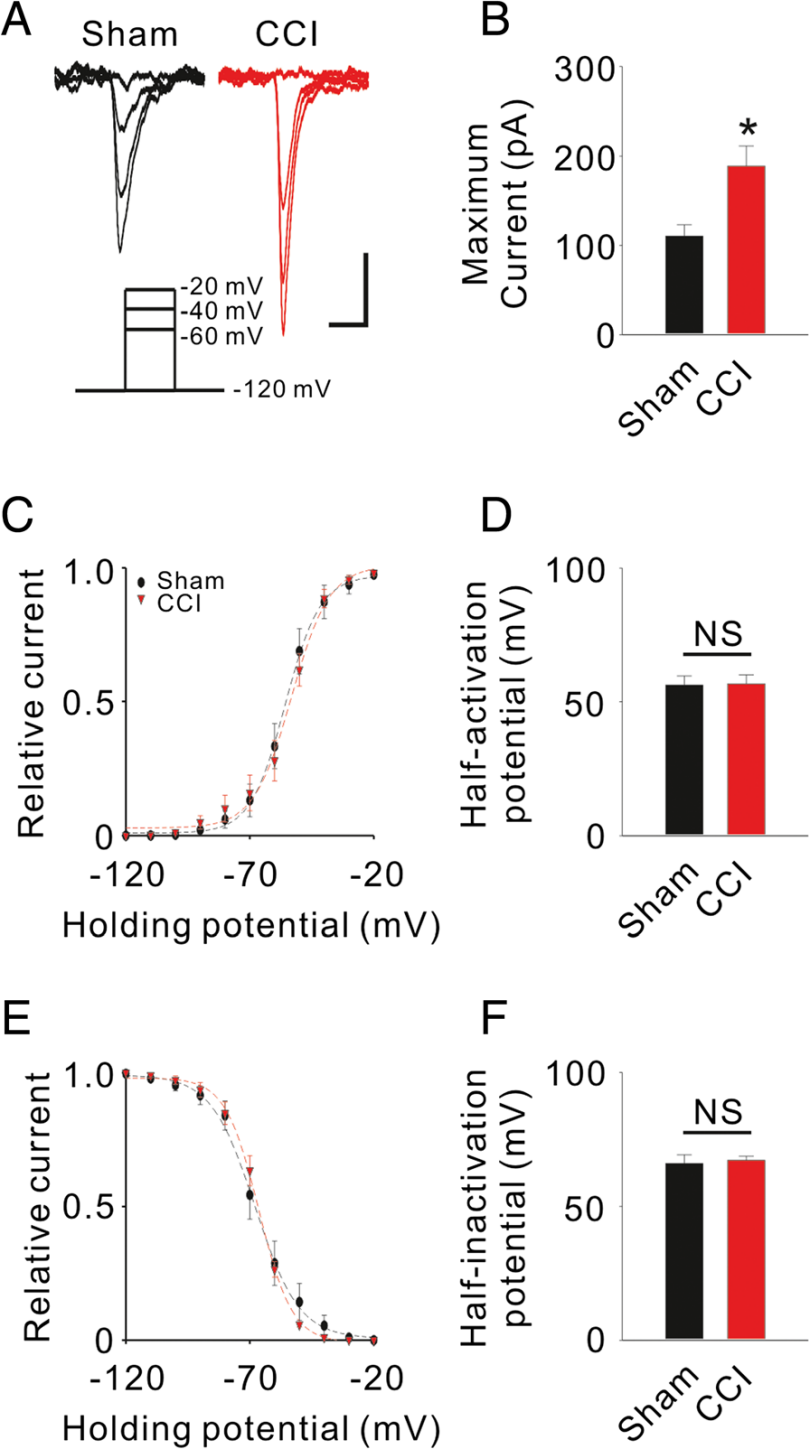
**Effect of CCI on intrinsic properties of TCCs in the ACC. A**, Induction of T-type currents with ascending depolarization from a sham (black) animal and a CCI (red) animal. Scale bars: 50 pA/80 mV and 50 ms. **B**, Comparison of maximum T-type current between the Sham and CCI groups. *P < 0.05 compared to the Sham group. **C**, Steady-state activation of T-type currents recorded from ACC pyramidal cells. **D**, Comparison of half-activation potential of TCC between the Sham and CCI groups. NS, not significant. **E**, Steady-state inactivation of T-type currents recorded from ACC pyramidal cells. **F**, Comparison of half-inactivation potential of TCC between the Sham and CCI groups.

We next compared the intrinsic properties of TCCs between sham animals and CCI animals. We found that the T-type currents were greater in the CCI group (Sham: *I*_*max*_ = 110.1 ± 12.83 pA, n = 12 vs. CCI: *I*_*max*_ = 188.8 ± 22.54 pA, n = 15, *P* = 0.016 using the normal Student’s *t* test; Figure 2A, B), and this result was consistent with the upregulation in TCC expression. However, the activation and inactivation kinetics were not significantly different between the two groups (Figure 2C-F). These data indicate that TCCs are normally embedded in the membrane of ACC neurons and that Ca_v_3.2 was upregulated after CCI surgery.

### Synaptic transmission in the ACC was attenuated by the TCC inhibitor

**Figure 3:**
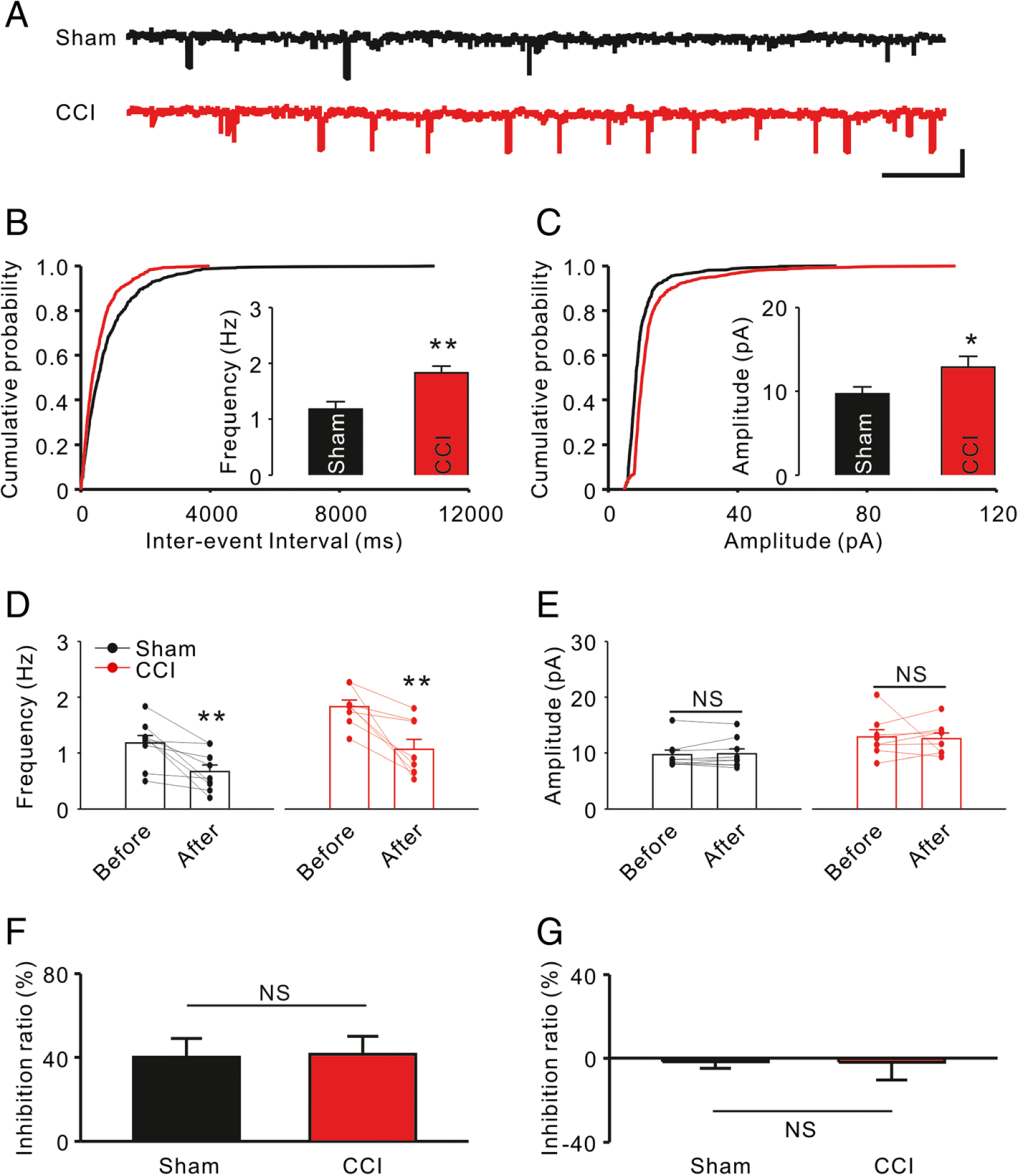
**Effect of TCC inhibitor on synaptic transmission of the ACC. A**, Representative mEPSCs recorded in ACC neurons from Sham (black) and CCI (red) groups at a holding potential of −70 mV. Scale bars represent 20 pA and 1 sec. **B**, Comparison of frequency of mEPSCs between the Sham and CCI groups. **P < 0.01 compared to the Sham group. **C**, Comparison of amplitude of mEPSCs between the Sham and CCI groups. *P < 0.05 compared to the Sham group. **D**, Effect of *NNC 55–0396* (20 μM) on frequency of mEPSCs. **P < 0.01 compared to the values before drug application. **E**, Effect of *NNC 55–0396* on amplitude of mEPSCs. NS, not significant. **F**, Inhibition ratio of *NNC 55–0396* on frequency of mEPSCs. **G**, Inhibition ratio of *NNC 55–0396* on amplitude of mEPSCs.

Xu et al. have demonstrated that both pre- and postsynaptic transmission was potentiated in the ACC after CCI surgery [6]. Further, such synaptic plasticity is responsible for generating neuropathic pain [7]. To confirm whether the TCC inhibitor affects synaptic transmission in the ACC, we investigated the amplitude and frequency of miniature excitatory postsynaptic currents (mEPSCs) in the brain slices before and after *NNC 55–0396* application. Consistent with previous studies, significant enhancements were found in the frequency (Sham: n = 9, 1.2 ± 0.13 Hz vs. CCI: n = 8, 1.8 ± 0.12 Hz, *P* = 0.003 using the normal Student’s *t* test; Figure 3A-B) and the amplitude (Sham: 9.7 ± 0.83 pA vs. CCI: 12.9 ± 1.29 pA, *P* = 0.03 using the Mann–Whitney Rank Sum Test; Figure 3C) of mEPSCs in ACC neurons after CCI surgery. *NNC 55–0396* significantly reduced the frequency (Sham: n = 9, 1.2 ± 0.13 Hz vs. 0.7 ± 0.12 Hz, *P* = 0.004 using the normal paired *t* test; CCI: n = 8, 1.8 ± 0.12 Hz vs. 1.1 ± 0.18 Hz, *P* = 0.005 using the normal paired *t* test; Figure 3D) but not the amplitude (Figure 3E) of the mEPSCs in both Sham and CCI groups. However, there were no significant differences in the inhibition ratio of frequency or in the amplitude between Sham and CCI groups (Figure 3F, G). These results indicate that the TCC inhibitor regulates presynaptic transmission more than postsynaptic transmission and that there is no difference between the Sham and CCI groups.

### ACC neuronal activity was reduced by the TCC inhibitor

**Figure 4:**
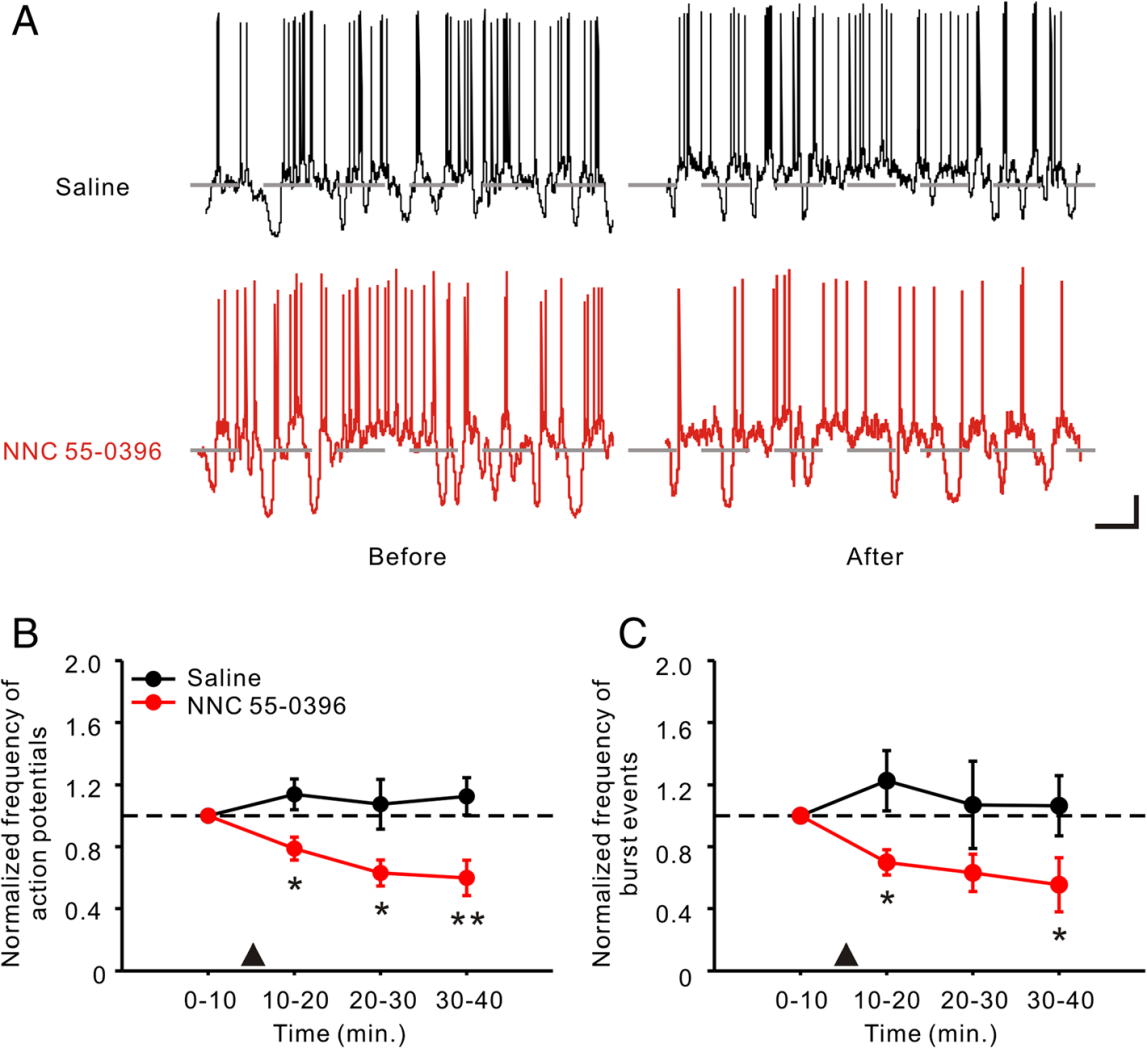
**Effect of TCC inhibitor on neuronal activity of ACC pyramidal cells in CCI rats. A**, Voltage traces showing the firing of ACC pyramidal cells in Saline (black) group and *NNC 55–0396* (red, 0.1 μg/kg) group before and 30 min after drug application by in vivo patch-clamp recording. Gray dash lines indicating the potential level of −60 mV. Scale bars: 10 mV and 1 sec. **B**, Effect of *NNC 55–0396* focal application on frequency of action potentials in ACC pyramidal cells. The frequency of action potential was sorted out from every time frame of 10 min. The drug application (▲) was performed after 10 min base recording. Data are presented over time as normalized values. *P < 0.05, **P < 0.01 compared to the Saline group. **C**, Effect of *NNC 55–0396* focal application on frequency of burst events in ACC pyramidal cells. *P < 0.05, compared to the Saline group.

Our previous work revealed that the neuronal activity in the ACC was facilitated after CCI surgery [8]. In the current study, we found that focal application of TCC inhibitor could significantly reduce the action potential frequency of ACC pyramidal cells in CCI rats (Saline: n = 6 vs. *NNC 55–0396*: n = 6, overall *P* < 0.001 using two-way ANOVA followed by Bonferroni’s *t*-test; Figure 4A, B). This change in the frequency of action potentials accompanied the reduction in burst events (overall *P* = 0.002 using two-way ANOVA followed by Bonferroni’s *t*-test; Figure 4C).

### Microinjection with TCC inhibitor relieves mechanical and thermal allodynia

**Figure 5:**
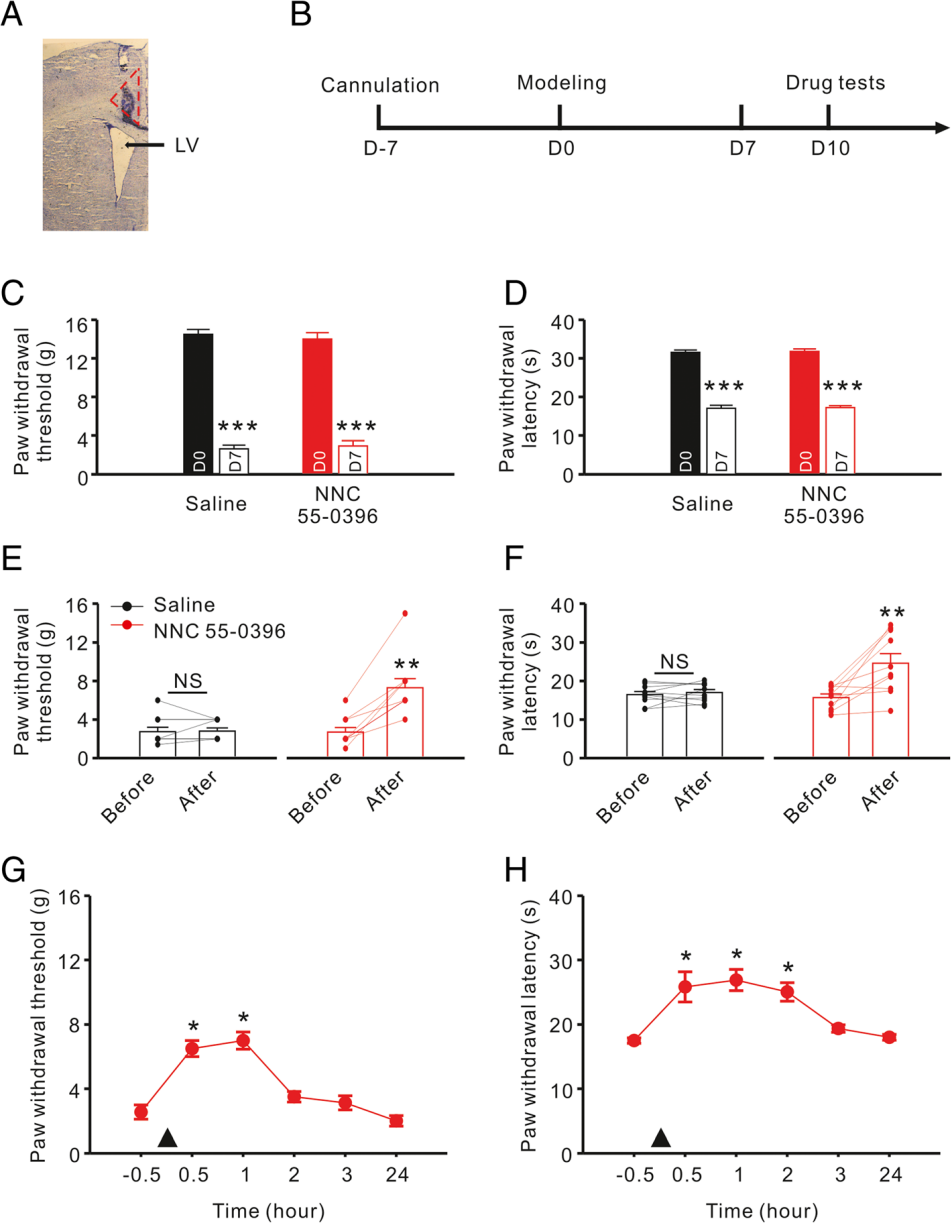
**Effect of ACC microinjection with TCC inhibitor on mechanical and thermal allodynia. A**, An example of microinjection site in an ACC slice with hematoxylin and eosin staining. The area in red dashed line indicating ACC, and the black arrow indicating lateral ventricle (LV). **B** *,* Experimental time schedule. D0 refer to the day of CCI surgery. **C**-**D**, Assessing the effect of CCI surgery. The stuffed bars representing the values before CCI surgery, whereas the hollow bars representing the values on D7. ***P < 0.001 compared to the values before CCI surgery. **E**-**F**, Comparison of the effect of Saline and *NNC 55–0396* (0.1 μg/kg) microinjection on mechanical and thermal allodynia. Black symbol representing the Saline group, and red symbol representing *NNC 55–0396* group. The drug effect was investigated 30 min after ACC microinjection on D10. **P < 0.01 compared to the values before ACC microinjection. NS, not significant. **G**-**H**, Effective drug duration of *NNC 55–0396* single treatment on mechanical and thermal allodynia. Horizontal ordinate representing the relative time of ACC microinjection. ▲: Drug application. *P < 0.05 compared to the values before microinjection.

To assess whether inhibiting TCCs in rat ACC could alleviate neuropathic pain, we compared the thresholds of mechanical and thermal allodynia before and after ACC microinjection with *NNC 55–0396*. First, we confirmed the microinjection site with hematoxylin and eosin staining (Figure 5A). One week after cannulation, CCI surgery was performed (the day of surgery is considered D0), and tests of the threshold of mechanical and thermal allodynia on D7 demonstrated that the modeling was successful (Figure 5B). Then, we divided the animals into equal groups for Saline and *NNC 55–0396* treatment (Figure 5C, D). The drug tests were performed on D10, when the mechanical and thermal allodynia had formed stably. *NNC 55–0396* significantly relieved the mechanical (Saline: n = 10, 2.7 ± 0.46 vs. 2.8 ± 0.33, *P* = 0.750 using the paired *t* test followed by Wilcoxon Signed Rank Test; *NNC 55–0396*: n = 10, 2.7 ± 0.47 vs. 7.3 ± 0.94, *P* = 0.002 using the paired *t* test followed by Wilcoxon Signed Rank Test; Figure 5E) and thermal (Saline: n = 10, 16.5 ± 0.79 vs. 17.0 ± 0.77, *P* = 0.473 using the normal paired *t* test; *NNC 55–0396*: n = 10 15.7 ± 0.95 vs. 24.6 ± 2.50, *P* = 0.004 using the normal paired *t* test; Figure 5F) allodynia 30 min after ACC microinjection. We also observed that the analgesic effects of *NNC 55–0396* were temporary: the thresholds of mechanical and thermal allodynia reverted to the base values on D14 (data not shown). To clarify the effective drug duration of *NNC 55–0396*, we tested the thresholds of mechanical and thermal allodynia over the course of 24 hours. The thresholds of mechanical (−0.5 h: 2.6 ± 0.44, n = 8 vs. 1 h: 7.0 ± 0.54, n = 8, P < 0.001 using one-way ANOVA followed by the Holm-Sidak post hoc test; Figure 5G) and thermal (−0.5 h: 17.5 ± 0.42, n = 8 vs. 1 h: 26.9 ± 1.65, n = 8, P < 0.01 using one-way ANOVA followed by Dunn’s Method; Figure 5H) allodynia both peaked at 1 h after ACC microinjection, and they both reverted to the base values within 3 h.

## Discussion

Todorovic and colleagues have demonstrated that CCI-induced neuropathy significantly increases T-type current expression in small dorsal root ganglion (DRG) neurons [15]. Moreover, intraperitoneal or intrathecal administration of TCC inhibitors has been shown to relieve the neuropathic pain [17-20]. TCCs are also expressed in some of the pain-associated brain regions, such as the thalamus and hypothalamus [21,22]. Therefore, the effects of systematical administration of TCC inhibitors on those brain regions cannot be ignored because some TCC inhibitors, such as ethosuximide, which is approved for clinical treatment of absence seizure, can pass through the blood–brain barrier [23]. Chen et al. have demonstrated that intracerebroventricular injection of TCC inhibitors could alleviate acid-induced chronic muscle pain [24]. These results suggest that modulating brain TCCs for treating neuropathic pain is feasible.

More and more evidence has shown that the ACC plays an important role in neuropathic pain [25-29]. We therefore speculated that modulating TCCs in ACC might alleviate neuropathic pain. In the current study, we found that at least two subunits (Ca_v_3.2 & Ca_v_3.3) of the TCC are present in the ACC and that the RNA level of Ca_v_3.2 was upregulated in a CCI model. This is consistent with the standpoint that Ca_v_3.2 supports the development and maintenance of both physiological and pathological pain [30]. Moreover, we found that that the T-type current intensity was increased when the CCI model was established stably. However, the activation and inactivation kinetics did not change significantly. These results are also consistent with reports of the studies on the peripheral nervous system [15]. Our results indicate that TCCs in the ACC may contribute to the maintenance of neuropathic pain.

Recent studies have demonstrated that TCCs regulate presynaptic neurotransmitter release [30-32], and our results support this standpoint. Because ACC synaptic plasticity is responsible for neuropathic pain generation [7], it is reasonable to use TCC inhibitors for pain management. However, Kang et al., using multi-electrode array recordings, reported that TCC had no contribution to the glutamatergic excitatory synaptic transmission in the ACC [13]. Kang’s study revealed that the contribution of TCCs in the ACC was no more than 5% in normal animals, whereas our study found that for all conditions, the contribution of TCCs was approximately 40% in presynaptic transmission. This discrepancy may arise from the differences in the study design.

Beacause our previous work demonstrated that the neuronal activity in the ACC is upregulated with CCI [8], we therefore supposed that pain perception might be reduced by inhibiting the neuronal activity of the ACC. First, we demonstrated that local application of TCC inhibitors could suppress the neuronal activity of the ACC in vivo. Then, we tested the effect of the drug on pain behavior. As expected, both mechanical and thermal allodynia were partially relieved by ACC microinjection with a TCC inhibitor, and the effect was temporary (<3 h). These results are indicating that neuropathic pain may be alleviated by inhibiting the activity of the ACC through modulating the TCCs.

Some limitations of the current study must also be considered. For T-type currents and synaptic transmission, we found the effect of the TCC inhibitor did not differ between the Sham and CCI groups. This result indicates that the pain threshold in both sham and CCI animals may be regulated by TCC inhibitor. However, the finding that TCC inhibitors may affect the pain perception in normal condition does not preclude the fact that ACC microinjection with TCC inhibitors relieves mechanical and thermal allodynia.

## Conclusions

Considering our results, we suggest that TCCs in the ACC may contribute to the maintenance of neuropathic pain and systemic administration of TCC inhibitors may have a partial effect on painassociated brain regions (e.g., the ACC) for the treatment of neuropathic pain.

## Methods

### Animals and modeling

Sprague–Dawley rats aged 9–11 weeks and weighing 250–350 g (Shanghai Sipper–BK Laboratory Animals Co., Ltd, Shanghai, China) were raised in cages at 24°C and 50%-60% humidity in a 12 h/12 h light/dark cycle with sufficient food and water supplied. All surgical procedures were performed under anesthesia with intraperitoneal injection of pentobarbital sodium (40 mg/kg). The research protocol was approved by the Animal Use and Care Committee for Research and Education of Shanghai Jiao Tong University. The number of animals used in this study met the minimum requirement for the purpose of the study.

The chronic constriction injury (CCI) model was established in accordance with the method described by Bennett [33]. In brief, the left sciatic nerves were exposed unilaterally after skin incision at the mid-thigh level and blunt dissection at the biceps. Four chromic gut (5–0) ligatures were tied loosely around the nerve at a 1–mm interval, proximal to its trifurcation. The day of surgery is considered day 0 (D0). The animals with paw withdrawal thresholds < 8 g or paw withdrawal latency < 20 s on D7 were considered successful for modeling neuropathic pain. Sham surgery was performed by exposing the sciatic nerve without ligation. The animals were used for experiments from D7 to D14.

### Mechanical and thermal allodynia test

For mechanical allodynia test, the animals were habituated for 2–3 days in the test environment before each test. The rats were placed in a plexiglass box with a metal net bottom for 30 min. After habituation to the environment, the hind paw was stimulated with one of a series of von Frey hairs with logarithmically increasing stiffness (0.6, 1, 1.4, 2, 4, 6, 8, 10, and 15 g) (Stoelting), presented perpendicular to the plantar surface (5–6 s for each hair). A positive performance was recorded when the rat escaped the mild pressure or raised the hind leg. Dixon’s up–down method was used to determine the 50% withdrawal threshold [34].

The thermal allodynia test used a different group of rats from the mechanical allodynia test. The rats were placed in a plexiglass box on a 3-mm-thick glass plate. After habituation in the box for 30 min, the sole skin of the animal was irradiated with light within a circle of 0.5 cm in diameter using a BME–410 thermal radiation stimulator (Peking Union Medical College Institute of Biomedical Engineering, China) at 10 V and 30 W. The time from the initiation of irradiation to paw withdrawal was recorded as the paw withdrawal latency value. Forty seconds was used as the cut-off time to avoid local burn injury. Three measurements were taken for each animal, with a 6–8 min interval, and the mean value was used for analysis.

### Quantitative real-time PCR

Total RNA from ACC tissues was extracted using TRIzol (Takara Biotechnology Co., Ltd.). The isolated RNA was subsequently treated with RNase-free DNase to remove genomic DNA contamination. cDNA was synthesized according to the manufacturer’s instructions (Takara Biotechnology Co., Ltd.). Primers (Table 1) were designed using Oligo 6 Primer Analysis software (Molecular Biology Insights Inc., Cascade, CO, USA). Each cDNA was amplified with SYBR® *Premix Ex Taq*™ Kit (Takara Biotechnology Co., Ltd.). The cycling conditions were 35 cycles of denaturation at 95°C for 30 seconds, primer annealing at 56°C for 30 seconds and primer extension at 72°C for 24 seconds. Each sample was analyzed in triplicate. Target gene expression was normalized against GAPDH expression levels and quantified using the 2^−ΔΔ*Ct*^ method.

**Table 1:** **Primer sequences of** β**-actin, Ca**_**v**_ **3.1, Ca**_**v**_ **3.2 and Ca**_**v**_ **3.3**

**Gene**	**Primer sequences**	**Product size (bp)**
GAPDH		194
Forward	5’- TATCGGACGCCTGGTTAC -3’	
Reverse	5’- GGAAGATGGTGATGGGTTT -3’	
Ca_v_3.1		344
Forward	5’-CTCCCAGATGCCCATCGGAG-3’	
Reverse	5’-AGGCATGACATGGTCAGC-3’	
Ca_v_3.2		287
Forward	5’-GAGTGTGCCTTGCCCCCTG-3’	
Reverse	5’-GGTGGCCTATCCCTCCTG-3’	
Ca_v_3.3		363
Forward	5’-CCATCAGCGTAGCCACAGCA-3’	
Reverse	5’-GCTGAGGAGCCCAAGCCT-3’	

### In vitro patch-clamp recordings

Brain slices were prepared in a manner similar to the one described by Xu et al. [6]. In brief, the animal was anesthetized with sodium pentobarbitone, and the brain was quickly excised from the skull and submerged in ice-cold artificial cerebrospinal fluid (ACSF, in mM: 119 NaCl, 1.3 MgSO_4_, 2.5 KCl, 1 NaH_2_PO_4_, 26.2 NaHCO_3_, 2.5 CaCl_2_, and 11 D-glucose). After being chilled for 1–2 min, the brain was trimmed to a block containing the ACC. With the use of a vibro-slicer (LEICA VT1000), coronal slices (300–450 μm) were cut from the tissue block in ice-cold ACSF. The slices were transferred to a gas-interface recording chamber that was perfused with aerated (95% O_2_/5% CO_2_) ACSF, at a rate of 0.5–1 ml/min, by a peristaltic pump-driven or gravity-fed bath-perfusion system at room temperature (22°C). Constant temperature (22°C), humidified 95% O_2_/5% CO_2_ gas mixture was continuously blown over the slices to further ensure adequate oxygenation of cells in the tissue.

Current- or voltage-clamp recordings were obtained from pyramidal cells in layer II/III of the ACC slices after equilibration for 1–2 h in the recording chamber. Whole cell recordings (series resistances: 15–22 MΩ) of TCCs were obtained with micropipettes (tip diameter: 1.5-2.0 μm; resistance: 4–5 MΩ) that were filled with an internal solution composed of (in mM): 140 K-gluconate, 10 HEPES, 2 MgCl_2_, 1 CaCl_2_, 11 EGTA, 2 K_2_ATP, pH 7.3. Normal ACSF, containing (in μM) 30 bicuculline methiodide, 2 nimodipine, 3 ω-conotoxin MVIIC, 0.5 tetrodotoxin, was used as the external solution in studying T-type currents [35]. The voltage errors resulting from the series resistance were compensated offline for voltage-clamp recordings and online for current-clamp recordings by using the bridge circuit. The signals from neurons were amplified with an Axoclamp-700B amplifier (bandwidth filter set at 10 kHz for current-clamp and 1 kHz for voltage-clamp recordings) and then digitized and sampled at 50-μs intervals (Digidata 1440A, pClamp 10.2; Molecular Devices).

The activation/inactivation kinetic curves were fitted with the Boltzmann equation: *I/I*_max_ = 1/{1 + exp[(*V – V*_*1/2*_)/*k*]}, where *I*_*max*_ is the maximum current obtained, *V*_*1/2*_ is the half-activation/inactivation potential and *k* is the steepness constant.

### In vivo patch-clamp recording

Animals were initially anesthetized with urethane (1.2 g/kg; intraperitoneally). After tracheotomy, the head was restrained in a stereotaxic apparatus (David Kopf Instr.) with the body temperature maintained at 37.3-37.8°C. For recording, a small craniotomy (~2 mm in diameter) was made above the left cortex, and a small piece of dura mater was carefully removed. Electrophysiological recordings were conducted at a light anesthesia level just below the threshold of body movements consisting of licking or scratching.

The micropipettes (tip diameter: 2.5-3.0 μm; resistance: 3–4 MΩ) were filled with an internal solution containing (in mM) 136.5 K-Gluconate, 17.5 KCl, 9.0 NaCl, 1.0 MgCl_2_, 10.0 HEPES, 0.2 EGTA, and Amphotericin B (0.5 mg/ml). The pH of the internal solution was adjusted to 7.3. Signals were acquired with an Axoclamp-200B amplifier, and sampled at 5 or 10 kHz using a Digidata 1440A with 1, 2, or 5 kHz low-pass filtering. The position of the ACC region was fixed by the stereotaxic coordinates (from Bregma, 1.2-2.8 mm anterior, 0.2-0.6 mm lateral; 1.5-2.0 mm beneath the cortical surface). For ACC local infusion, glass pipettes full of saline or *NNC 55–0396* (0.1 μg/kg) with tip openings of ~50 μm were used, and they were placed within ~100 μm of the recording pipettes.

The action potentials were detected using Threshold Research, and the burst firings were detected using Burst Analysis. Minimum events in burst = 2, Burst delimiting interval = 100 ms (pClamp 10.2).

### Cannulation and microinjection

The rats were anesthetized with sodium pentobarbital and secured on the stereotaxic apparatus. A midline incision was made to expose the skull of the rat. Based on the position determined by the stereotaxic atlas, 28 gauge guide cannulas were bilaterally implanted in the ACC at the following coordinates: 2.7 mm anterior from bregma, ± 0.6 mm lateral to midline, and 2.0 mm ventral from the dura; 0.4 mm anterior from bregma, ± 0.6 mm lateral to midline, and 2.0 mm ventral from the dura. Around the guide cannulas, three stainless screws were implanted into the skull and fixed with dental acrylic. The dummy cannulas were inserted after surgery until the infusion day. CCI was performed immediately after cannulation. The animals were raised individually after surgery. The dummy cannulas were pulled out before microinjection. The internal injection cannulas (32 gauge), extending 1 mm beyond the guide cannulae, were inserted. Saline or *NNC 55–0396* (0.1 μg/kg) was injected at a constant rate for longer than 120 s with a micro-injector. The injection cannulas were left in place for an additional 3 min before being withdrawn. The animals were allowed to move freely during the entire injection process. An additional 30 min was given before the beginning of behavioral test. Microinjection was performed on D10.

### Drugs

All drugs were purchased from Sigma, and they were diluted from the stock solutions to the final desired concentration in the ACSF or saline immediately before use.

### Statistical analysis

Numerical data are expressed as the mean ± S.E.M. Student’s *t* test was used for the comparison of two independent datasets with normal distribution, whereas the paired *t* test and Wilcoxon Signed Rank Test were performed for comparisons of two dependent datasets with and without normal distribution, respectively. One-way ANOVA and pairwise comparison with the Holm-Sidak test were performed to compare multiple independent datasets with normal distributions, whereas Kruskal-Wallis one-way ANOVA on ranks was performed to compare datasets without normal distributions. Two-way ANOVA with Bonferroni’s post hoc test was performed to determine wheter there was a contingency between the two kinds of classification. *P <*0.05 was considered significant.

## Abbreviations

ACC: Anterior cingulate cortex
TCC: T-type calcium channel
CCI: Chronic constriction injury
ACSF: Artificial cerebrospinal fluid
